# Whole Body and CNS Biodistribution of rhHNS in Cynomolgus Monkeys after Intrathecal Lumbar Administration: Treatment Implications for Patients with MPS IIIA

**DOI:** 10.3390/ijms18122594

**Published:** 2017-12-01

**Authors:** Jou-Ku Chung, Luying Pan, Kathleen Palmieri, Amir S. Youssef, Thomas G. McCauley

**Affiliations:** 1 Nonclinical Development, Research and Discovery, Shire, 300 Shire Way, Lexington, MA 02421, USA; joukuc@gmail.com (J.-K.C.); kzpalmieri@shire.com (K.P.); tmccauley000@gmail.com (T.G.M.); 2 KinderPharm/PKPD Bioscience Inc., 100 Arrandale Blvd #101, Exton, PA 19341, USA; ayoussef@kinderpharm.com

**Keywords:** enzyme replacement therapy, recombinant human heparan *N*-sulfatase, mucopolysaccharidosis III type A, Sanfilippo syndrome, lysosomal disorder, intrathecal administration, positron emission tomography, pharmacokinetics, cerebral spinal fluid

## Abstract

Mucopolysaccharidosis III type A (MPS IIIA; Sanfilippo syndrome), a genetic lysosomal disorder causing a deficiency of heparan *N*-sulfatase (HNS), leads to progressive cognitive decline from an early age. An effective enzyme replacement therapy (ERT) for MPS IIIA requires central nervous system (CNS) biodistribution. Recombinant human heparan *N*-sulfatase (rhHNS), an investigatory ERT for MPS IIIA, has been formulated for intrathecal (IT) administration since intravenous (IV) administration cannot cross the blood brain barrier (BBB) in sufficient amounts to have a therapeutic effect. In this study, systemic and CNS distribution of rhHNS in cynomolgus monkeys following IV and IT administration was evaluated by quantitation of rhHNS in serum, cerebral spinal fluid (CSF) and various tissues, and positron emission tomography (PET) imaging of live animals. Following IV administration, rhHNS levels were low to non-detectable in the CSF, and systemic clearance was rapid (≤2 h). With IT administration, rhHNS was observable in CNS tissues in ≤1 h, with varying T_max_ (1–24 h). Appreciable systemic distribution was observed up to 7 days. This provides evidence that in this animal model, intrathecal administration of rhHNS delivers the replacement enzyme to therapeutically relevant tissues for the treatment of Sanfilippo Syndrome type A. Penetration into grey matter and cortex was 3–4 times greater than concentrations in white matter and deeper parenchymal regions, suggesting some limitations of this ERT strategy.

## 1. Introduction

Sanfilippo syndrome type A, also called mucopolysaccharidosis type IIIA (MPS IIIA), is an autosomal recessive lysosomal disorder that causes a deficiency in the enzyme heparan *N*-sulfatase (HNS) [1]. MPS IIIA primarily manifests in the central nervous system (CNS) and brain. Consequent buildup of glycosaminoglycan (GAG)/heparan sulfate (HS) causes progressive neurodegeneration in affected children, with symptoms presenting in the second year of life. The cognitive development of affected children is marked by delays, arrest, and ultimately regression [2]. In addition, their cognitive decline is accompanied by increasing behavioral and sleep disturbances, motor impairment, and, in some cases, seizures. The lifespan of children with MPS IIIA typically does not extend beyond late teens to early twenties [2,3].

Recombinant human heparan *N*-sulfatase (rhHNS) is an investigatory enzyme replacement therapy (ERT) for the treatment of MPS IIIA. Intravenous (IV) delivery of rhHNS has not demonstrated appreciable CNS penetration needed for therapeutic treatment of the neurocognitive degeneration [4]. Formulation of rhHNS for intrathecal (IT) administration allows direct delivery to the CNS via the cerebral spinal fluid (CSF). Evidence of the tolerability of chronic intrathecal administration of rhHNS was initially provided in a device- and vehicle-controlled safety evaluation in cynomolgus monkeys dosed every two weeks over six months at three dose levels [4]. In a subsequent open-label, phase 1/2 dose-escalation, safety trial in 12 human patients for a total of six doses, rhHNS administered via an intrathecal drug delivery device (IDDD) was generally well tolerated [5]. Jones, et al., reported that treatment with rhHNS resulted in consistent declines of HS in CSF over six months of treatment. 

The utility of IT ERT dosing for cognitive efficacy has been previously evaluated for the treatment of patients with Mucopolysaccharidosis II (MPS II, Hunter Syndrome), which causes progressive cognitive decline in 60–80% of patients [6,7]. An IT formulation of the enzyme iduronate-2-sulfatase (idursulfase IT) dosed in cynomolgus monkeys showed appreciable and dose-dependent levels of idursulfase IT in the CNS and in serum [8,9,10]. In humans, idursulfase IT administered via IDDD in a Phase I/II trial showed marked reduction in CSF GAG among patients with Hunter Syndrome, and the treatment is currently being tested in a Phase II/III clinical trial for young patients with early cognitive impairment (NCT02055118) [6].

We report the findings of two separate studies that assessed the biodistribution of rhHNS following IT and IV administration in cynomolgus monkeys. Pharmacokinetic parameters were analyzed to evaluate the rhHNS biodistribution and compare its distribution between dose routes and anatomical and/or systemic compartments. The maximal or peak concentration achieved after administration (C_max_) and time this peak concentration occurs at, T_max_, the area under the concentration curve, or AUC, for the time points tested (AUC_0-last_) and extrapolated to infinity (AUC_inf_) and the terminal half-life (T_1/2_) derived from these curves were calculated to more fully characterize the dynamic distribution behavior of IV and IT dosed rhHNS. 

The pharmacokinetics and tissue penetration profiles of animals dosed with rhHNS was assessed by serum and CSF sampling at specified time points (Study A—phase 1 and Study B), by animal sacrifice and tissue harvest at specific time points (Study A—phase 2), and by positron emission tomography (PET) imaging using 124-iodinated (^124^I) rhHNS at specific time points (Study B) (Table 1). In Study A, the HNS concentration in serum and CSF was measured using an enzyme linked immunosorbent assay (ELISA) and the HNS activity in liver, brain, and spinal cord tissue homogenates were determined using an HNS enzymatic activity assay. Study B visualized the dynamic uptake of rhHNS from the CSF to the CNS tissue, and between the CNS and systemic circulation, using ^124^I radio-labelled rhHNS. Following IV administration, rhHNS levels in CSF and CNS tissues were low to non-detectable, indicating the integrity of the blood brain barrier (BBB) toward the therapeutic enzyme. IT dosing resulted in rapid uptake of rhHNS from CSF into CNS tissues and appreciable systemic distribution to the serum and peripheral organs. These data support intrathecal delivery methods for ERTs targeting CNS as well as systemic distribution.

## 2. Results

### 2.1. IT Dosing of rhHNS Yields Longer Term Detectable Signals in the CSF and Serum Compared to the Rapid Decline of rhHNS Signals in Serum Dosed IV

**Figure 1:**
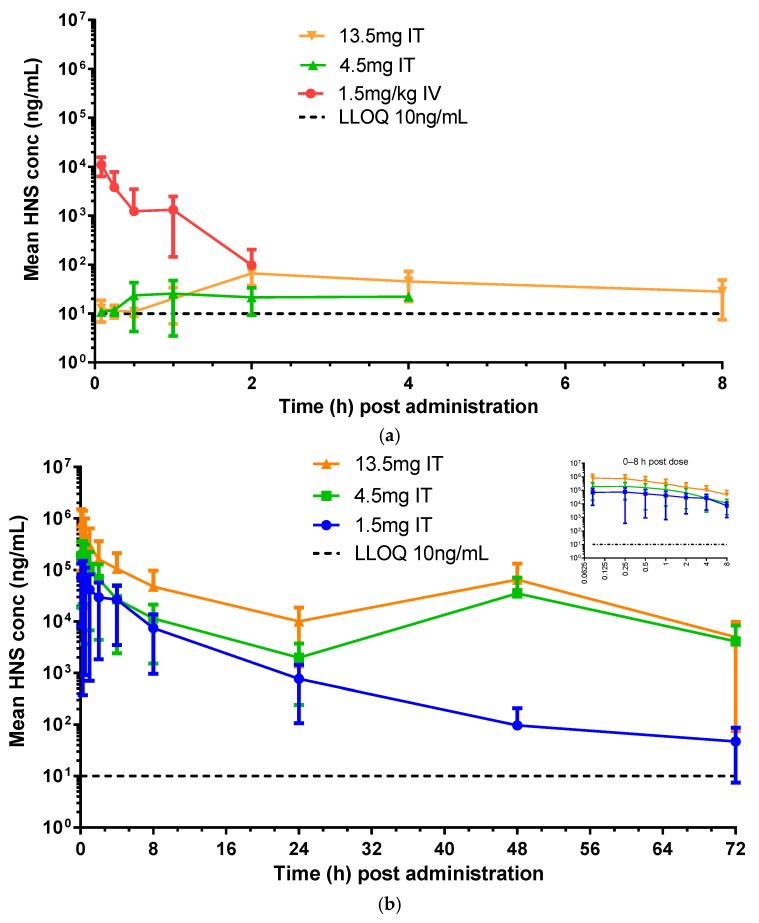
Levels of rhHNS dosed intravenous (IV) and intrathecal (IT) at specified time points in six animals per dose as determined by enzyme linked immunosorbent assay (ELISA). (**a**) in serum (**b**) in cerebral spinal fluid (CSF) Note: Serum heparan *N*-sulfatase (HNS) was below the limit of quantification (BLQ) at all time points for the IT 1.5 mg dose group and is not shown in Figure 1a. Serum HNS was BLQ after the 2 h time point for the IV 1.5 mg/kg dose group in Figure 1a. CSF HNS was BLQ for a majority of animals and time points for the IV 1.5 mg/kg dose group therefore data is not shown in Figure 1b.

CSF and blood samples were collected at specific time points after IV and IT dosing of rhHNS in six animals per dose group for HNS quantification by ELISA. In Study A, where monkeys received IV rhHNS at 1.5 mg/kg by a slow bolus via a peripheral vein, the peak serum concentration (mean ± SD) was 11,082 ± 4712 ng/mL at 5 min after dosing, and the last time point with measurable HNS in serum was at 2 h post dose with a concentration of 97.8 ± 105 ng/mL (detectable in two animals) (Figure 1a). CSF concentrations for this dose were below the limit of quantification (BLQ) for three animals across all time points and BLQ or less than the lower limit of quantification (LLOQ = 10 ng/mL) for seven of 10 time points for the remaining animals. HNS was last detectable in CSF, at 10.1 ng/mL in one animal at the 2 h time point. Due to the sparse data, mean HNS concentrations are not shown for the IV 1.5 mg/kg dose group in Figure 1b. The peak mean CSF concentration was 20.3 (8.74) ng/mL at 45 min post dose which was approximately 500 times lower than the peak mean serum HNS concentration. HNS concentrations fell to or below lower limit of quantitation in both serum and CSF by 2 h.

The HNS concentrations in serum and CSF from monkeys receiving rhHNS at 1.5, 4.5 & 13.5 mg (*n* = 6/group) via an intrathecal catheter installed surgically in the lumbar spine at L4, L5, or L6 are presented in Figure 1a,b. When rhHNS was IT administered at 1.5 mg, HNS serum concentrations were BLQ for all animals at all time points. The serum C_max_ of rhHNS for 4.5 and 13.5 mg IT dose groups were low: 31.7 ng/mL (no SD for two subjects only) and 76.6 ± 22.1 ng/mL at 2.5 h post dose, respectively. With the 4.5 mg dose, HNS was last detectable at 22.2 ng/mL (*n* = 1) at the 4 h. With the 13.5 mg dose, rhHNS was last detectable at 28.1 ± 20.6 ng/mL at 8 h (*n* = 3). In CSF, the C_max_ was 176,295 ± 60,547, 406,985 ± 235,356, and 1,618,967 ± 854,904 ng/mL for 1.5, 4.5, and 13.5 mg dosing group, respectively. The T_max_ was at 0.8 ± 1.4 h, 0.3 ± 0.3 h, and 0.4 ± 0.3 h for 1.5, 4.5, and 13.5 mg dosing group, respectively. The peak CSF exposures had halved by 1 h post dose and decreased gradually over 24 h. The 4.5 and 13.5 mg doses showed monotonic decreases in HNS concentration in the CSF from 0 to 24 h, a transient increase from 24 to 48 h (due to elevated concentrations in one animal in the 4.5 mg dose group and in two animals in the 13.5 mg dose group), and a decrease from 48 to 72 h. HNS was still measurable in the CSF in the majority (≥5) of dosed animals at 72 h post dose: 83.6 ± 52.6 ng/mL, 3891 ± 8425 ng/mL, and 5116 ± 9809 ng/mL in the 1.5, 4.5, and 13.5 mg groups, respectively.

### 2.2. Appreciable rhHNS Bioavailability and Dose Proportionality in CSF and Serum Post–IT Dosing Is Observed

**Figure 2:**
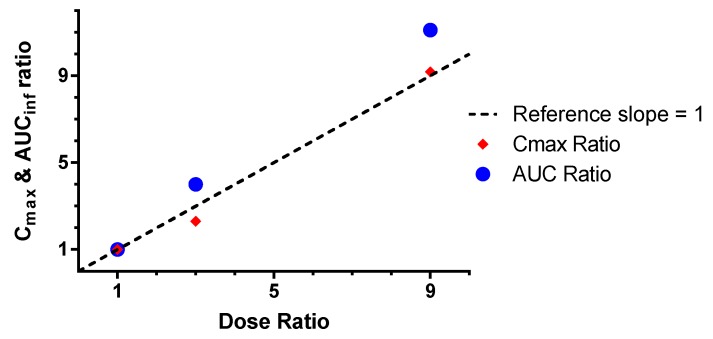
Dose proportionality in the CSF after IT dosing as measured by C_max_ and AUC_inf_ ratios.

The bioavailability of rhHNS was estimated for serum and CSF as dose normalized in the following way: F %( IT relative to IV) = (AUC_inf IT_/AUC_inf IV_) × 100 and F %( IV relative to IT) = (AUC_inf IV_/AUC_inf IT_) × 100. The dose-normalized bioavailability of rhHNS in the CSF after IV dosing was <0.003% of the exposure in CSF after IT administration for all doses (1.5, 4.5, and 13.5 mg). Conversely, the dose-normalized serum bioavailability of rhHNS after IT dosing was not detectable at 1.5 mg, yet was 7.01% and 4.78% for 4.5 and 13.5 mg, respectively. Both C_max_ and AUC_inf_ CSF values after IT dosing increased in an approximately dose-proportional manner (Figure 2).

### 2.3. Tissue Analysis after Intrathecal Administration Showed Early and Varying CNS Penetration

Selected tissues were harvested from animals dosed 14.5 mg rhHNS IT at selected time points for histopathology assessments of CNS penetration by enzyme activity analysis (Table 2 and Figures S1 and S2). Cellular distribution of rhHNS was detected by immunohistochemistry analysis of rhHNS in meningeal cells, glial cells, and neurons in the brain and spinal cord (data not shown). For the individually sectioned spinal cord tissues (cervical, thoracic, and lumbar), similar HNS activities were observed in tissue homogenates (data not shown). These results revealed no evidence of a gradient trend along the length of the spinal cord. Across CNS tissues, liver, and kidney at the sampled time points, HNS was detected within 1 h (the earliest time point sampled), individual peak concentrations were found from 1–24 h (means ranged from 2.2 h for brain nucleus tissues to 24 h for cerebral cortex), and HNS was still detectable at 7 days post dose in spinal cord slices −8 to 10 (BLQ for slices 12 and 14), the superficial cerebral cortex (superficial, frontal, and temporal), white matter deep (right), caudate nucleus, corpus callosum, hippocampus (right), cerebellum (right), kidney, and liver but BLQ in tissue samples of the thalamus and hypothalamus.

**Figure 3:**
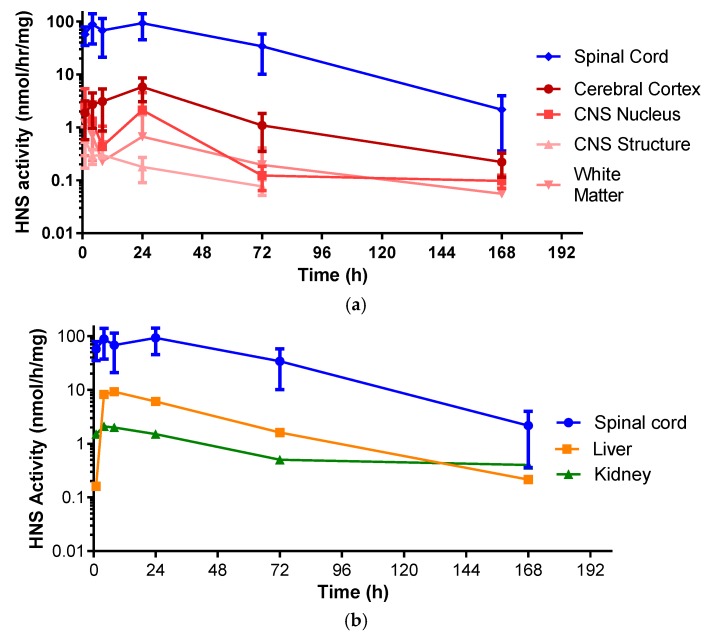
(**a**) Enzyme activity profiles of rhHNS in spinal cord and brain tissues from four animals after administration of IT rhHNS 14.5 mg. Activity profiles from tissue homogenates are expressed as rhHNS activity with the units nmol/h/mg. Cerebral Cortex (superficial, temporal, and frontal cortex); CNS Nucleus (caudate nucleus, hypothalamus, and thalamus); CNS Structures (cerebellum, corpus callosum, and hippocampus); White Matter (deep and superficial); (**b**) Enzyme activity profiles of rhHNS in spinal cord and peripheral organs from four animals after administration of IT rhHNS 14.5 mg. Activity profiles from tissue homogenates are expressed as HNS activity with the units nmol/h/mg.

HNS activity profiles from brain components of monkeys dosed with IT rhHNS 14.5 mg revealed similar peak exposure times and exposure half-lives in various tissues. The exposure across the analyzed brain tissues in Table 2 (cerebral cortex: superficial, temporal, and frontal cortex; CNS nucleus: caudate nucleus, hypothalamus, and thalamus; CNS Structures: cerebellum, corpus callosum, and hippocampus; and the white matter: deep and superficial) (0.6–5.8 nmol/h/mg protein) was more than 10-fold lower than in the spinal cord (83.2–133.0 nmol/h/mg protein across eight slices). Of the brain tissues, the cerebral cortex showed the greatest activity levels of HNS (C_max_ 5.83 nmol/h/mg protein). The CNS nucleus (caudate nucleus, thalamus, and hypothalamus), CNS structures (corpus callosum, hippocampus, and cerebellum), and the white matter showed roughly similar enzyme activity levels (C_max_ 0.635 nmol/h/mg protein, 2.56 nmol/h/mg protein, and 1.36 nmol/h/mg protein, respectively; Figure 3a and Figure S3).

**Figure 4:**
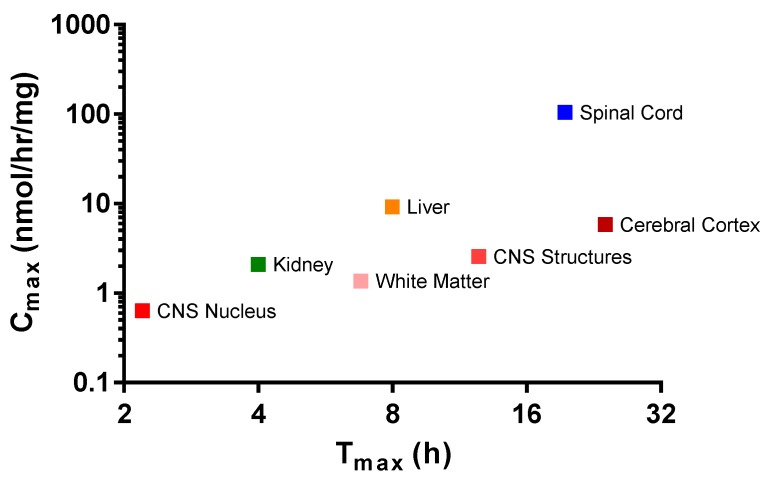
Mean peak exposures and times of rhHNS in sampled tissues after administration of IT rhHNS 14.5 mg. C_max_ values from tissue homogenates are expressed as HNS activity with the units nmol/h/mg. Cerebral Cortex (superficial, temporal, and frontal cortex); CNS Nucleus (caudate nucleus, hypothalamus, and thalamus); CNS Structures (cerebellum, corpus callosum, and hippocampus); White Matter (deep and superficial).

In animals who received IT rhHNS 14.5 mg, the peripheral organs, kidney, and liver had similar enzyme activity profiles, but liver exposure was four times greater than kidney exposure (C_max_ 9.2 vs. 2.1 nmol/h/mg protein, respectively). In both peripheral organs, HNS was first observed within 1 h post dose and was still observed at 7 days (Figure 3b). The relative peak exposures and times of HNS in sampled tissues after IT administration are shown in Figure 4.

### 2.4. ^124^I Radiolabelled rhHNS Concentrations after IT Dosing Suggest a Gradual, Controlled Uptake from CSF to Venous System

Radiolabeled rhHNS delivered to monkeys by IV bolus and by intrathecal lumbar injection enabled the estimation of pharmacokinetic parameters of replacement enzyme in systemic circulation. Blood samples taken at the start of each whole-body imaging study and analyzed using size exclusion HPLC to determine the disassociation of ^124^I radiolabel showed that the majority of radioactivity detected in the blood was rhHNS-bound. Following administration of IV rhHNS 1.0 mg/kg in four animals, the mean C_max_ (±SD; range) of rhHNS was 13,225 (±4470; 6747–16,850) ng/mL at a mean T_max_ (±SD; range) of 0.04 (±0.03; 0.03–0.08) h post dose. The rhHNS concentration decreased rapidly at the first h, suggesting a rapid clearance from the blood compartment. Following administration of IT rhHNS 3.0 mg in four animals, the C_max_ of rhHNS in the blood was 304 (±87; 210–390) ng/mL at a mean T_max_ of 30.75 (±22.16; 2.45–48.57) h post dose, suggesting a gradual, controlled distribution of rhHNS from the CSF to the venous system. The relative blood bioavailability of rhHNS following IT administration was estimated by the comparison of partial exposure, AUC_0–48_. Following the dose correction, the mean (±SD; range) blood bioavailability of rhHNS post IT administration was 47.6% (±8.6; range 38.1–73.6%). However, this estimate is from a truncated exposure profile and involves a dose correction, so the interpretation is limited.

### 2.5. PET Imaging Reveals Dynamic Distribution of IT-Dosed rhHNS from CSF to CNS to Systemic Peripheral Organs

**Figure 5:**
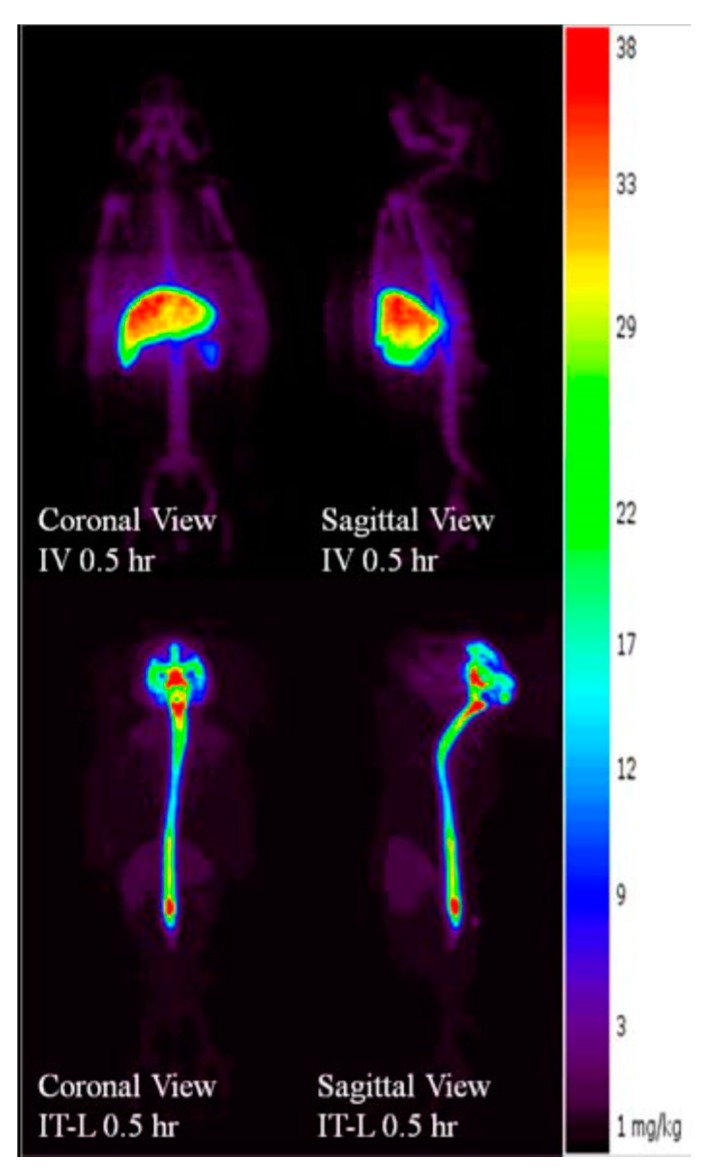
Positron emission tomography (PET) imaged biodistribution profiles of rhHNS 0.5 h following administration of intravenous (IV) rhHNS 1.0 mg/kg and intrathecal (IT) rhHNS 3.0 mg.

The radiolabeled enzyme and PET imaging of IV and IT dosed animals also enabled a dynamic assessment of the pharmacokinetics in the CNS and systemically. Animals dosed rhHNS IV 1.0 mg/kg showed non-detectable rhHNS levels in CNS and accumulation of rhHNS to varying extents in the major organs (liver, spleen, and kidneys; Figure 5) by PET imaging. In all systemic tissues, rhHNS reached C_max_ in a few h after IV administration, suggesting a rapid tissue distribution and uptake. The mean C_max_ (±SD) (ng/g) at T_max_ (±SD) (h) was 29,165 (±5034) at 2.3 (±1.8) in liver, 8876 (±2964) at 3.0 (±1.4) in spleen, 1755 (±543) at 8.4 (±10.6) in kidney, 2334 (±655) at 2.1 (±1.9) in heart, 1441 (±298) at 7.9 (±10.9) in lung, 10,286 (±3724) at 42.0 (±12.0) in stomach, and 4610 (±4069) at 24.0 (±0.0) in bladder. All systemic tissues exhibited similar mono-phasic elimination profiles with 1.0 mg/kg IV dosing. 

**Figure 6:**
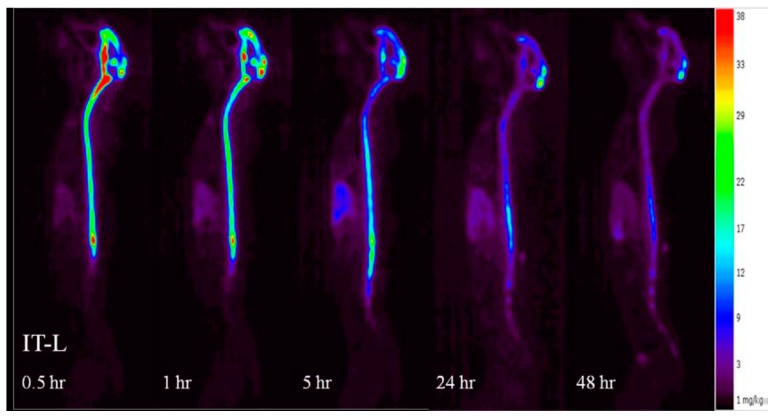
Whole-body positron emission tomography (PET) biodistribution kinetic images of rhHNS 0.5–48 h after administration of IT rhHNS 3.0 mg.

At 0.5 h post dose, animals dosed 3.0 mg IT showed clear radioactivity signals in the spinal cord and CNS tissues suggesting uptake from CSF to CNS within 30 min of IT administration (Figure 5). In general, rhHNS reached C_max_ in systemic tissues a few h after IT administration, suggesting rapid distribution from CSF to systemic circulation and quick tissue uptake (Figure 6). Following 3.0 mg IT dosing, the mean C_max_ (±SD) (ng/g) at T_max_ (±SD) (h) was 11,738 (±4911) at 9.8 (±9.5) in liver, 5378 (±2256) at 9.5 (±9.7) in spleen, 1140 (±484) at 4.3 (±0.5) in kidney, 1583 (±649) at 9.1 (±9.9) in heart, 960 (±379) at 8.8 (±10.2) in lung, 8085 (±1264) at 42.0 (±12.0) in stomach, and 2430 (±647) at 36.0 (±13.9) in bladder. All systemic tissues exhibited mono-phasic elimination profiles for 3.0 mg IT dosing. 

**Figure 7:**
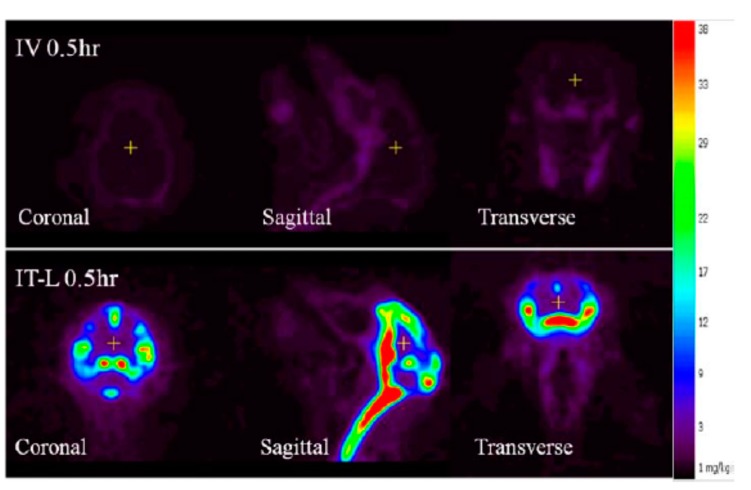
Positron emission tomography (PET) image of brain biodistribution of rhHNS at 0.5 h following administration of intravenous (IV) rhHNS 1.0 mg/kg and intrathecal (IT) rhHNS 3.0 mg.

Compared to the non-detectable levels of rhHNS in the brain 0.5 h after IV administration, approximately 90% (55% in whole brain and 35% in spinal cord) of 3.0 mg IT rhHNS was detected in the CNS tissue at that time point. At 0.5 h post-IT dose, rhHNS exposure in the brain was stronger in superficial regions than in the deeper parenchymal regions (Figure 7). Analysis of cerebral uptake and brain distribution of IT rhHNS 3.0 mg at 5 h (Figure S4) indicated clearance of rhHNS from the CSF ducts (septum pellucidum and third/fourth ventricles) and accumulation in the parenchyma and arachnoid. The high level of CNS exposure post IT administration of a 3.0 mg dose resulted in penetration of rhHNS in the grey and white matter (grey matter ~4× white matter; mean C_max_ 17,738 (9153) vs. 4455 (1649) ng/g). At 48 h, the radioactivity signals were still detectable in the spinal cord and brain (Figure 6).

## 3. Discussion

In MPS IIIA–affected children, GAG accumulation in the CNS, and specifically the brain and spinal cord, results in neurocognitive decline and early death. Development of an effective treatment of MPS IIIA, either by enzyme replacement therapy or viral gene transfer, is challenging, as both require delivery to or expression of HNS within the CNS, which is protected by the blood-brain barrier (BBB) [11,12]. A novel therapy with gene transfer vector using the adeno-associated virus serotype 9 (AAV9) was reported to efficiently cross the BBB following IV delivery in MPS IIIA and MPS IIIB mouse models [13,14]. Although the AAV9 vector has been shown to lead to a widespread expression of the transgene in the disease mice, the effect in crossing through BBB and improving CNS pathology in humans is yet to be proven [15,16,17]. The recent success of using substrate reduction therapy (SRT) with the aim to prevent storage but not to correct the enzymatic defect was reported [18,19]. Unfortunately, no improvement in the neurological manifestations was detected when patients received daily treatment [20,21]. Furthermore, the long term (5–9 years) clinical study for patients with Hunter syndrome using intravenous ERT showed significant improvement in somatic organs, however, no improvement in respiratory function or eye or CNS disease was found [22]. The development of rhHNS administered into the CSF via a surgically implanted IDDD for patients with MPS IIIA attempts to overcome the inability of this ERT to cross the BBB via IV administration. As the disease progresses, structural and functional damage to the highly integrated microvascular CNS barrier can cause impairment to both influx and efflux systems, thus adding further uncertainty to IV dosing strategies [23]. One advantage of delivering the ERT directly to the CSF space was described by Calias et al. in a review on protein therapeutics in the CSF [24]. These authors noted that cellular uptake of recombinant proteins by neurons and glial cells, which are mediated by mannose-6-phosphate receptors and additional uptake mechanisms, can reduce the overall clearance of protein therapeutics.

An open-label Phase I/II dose escalation safety trial of IT rhHNS in 12 patients with MPS IIIA dosed monthly for six months via an IDDD showed that rhHNS was generally safe and well tolerated and led to consistent declines in CSF HS indicative of functional enzymatic activity in the relevant CNS structures [5]. Although clinical safety and tolerability was observed, the study was not designed to evaluate the effects of IT rhHNS on disease progression. The experimental studies presented here provide more detailed information about the pharmacokinetics and biodistribution of IT rhHNS than would be possible in a clinical trial in human patients. 

Determining the extent of penetration beyond the site of injection, and in particular, the disposition of rhHNS in the cerebrospinal space with respect to the exposure of specific brain structures or cell types to rhHNS, is important in assessments of therapeutic potential or, conversely, the safety risks of treatment. Previous to the present studies, a safety and tolerability experiment with IT rhHNS in juvenile cynomolgus monkeys found a minimal increase in CNS cellular infiltrates which resolved within one month of the last dose and which were not associated with any adverse clinical signs, gross CNS lesions, or morphological changes in the brain or spinal cord [4]. A trend in dose-to-rhHNS CSF concentration and to rhHNS activity in CNS tissues was also demonstrated. 

In a similar study of monthly intrathecal administration of idursulfase (an ERT for MPS II, Hunter Syndrome) in cynomolgus monkeys, cellular penetration into relevant CNS tissues was demonstrated through immunohistochemical analysis using anti-human idursulfase monoclonal antibodies [8]. Specifically, 30-mg and 100-mg doses of idursulfase-IT localized iduronate-2-sulfatase (I2S) to neurons (deep neurons adjacent to white matter, cerebral neurons, meningeal, and glial cells in surface neurons next to the meninges), as well as to neurons, meningeal, and glial cells in the spinal cord. Idursulfase activity, measured by the fluorimetric substrate 4-Methylumbelliferyl-alpha-iduronate 2-sulphate, showed above baseline levels of I2S in the brain and spinal cord [25]. Calias et al., using PET imaging of ^124^I-labelled idursulfase, investigated the biodistribution of IT idursulfase in cynomolgus monkeys and found the presence of I2S in the brain parenchyma and the spinal cord [9]. Using anti-human idursulfase monoclonal antibodies, cellular deposition of I2S in the neurons of the cerebrum, cerebellum, brainstem, and spinal cord was dose-dependent and widespread. Surface gray matter, neurons of the thalamus, hippocampus, caudate nucleus, and spinal cord also showed I2S deposition. The pharmacokinetics and bioavailability of idursulfase IT in cynomolgus monkeys studied by Xie et al. showed that I2S C_max_ in the CSF was 8000–30,000 times greater than when it was dosed intravenously [10]. The long half-life observed in that study (approximately 10 h) suggests it may have therapeutic potential in elimination of GAG in the CNS [24]. These findings were confirmed in a Phase I/II trial in 16 patients with Hunter Syndrome [6]. In that trial, CSF GAG levels were significantly reduced, and a Phase 2/3 trial of idursulfase IT is underway (NCT02055118).

Results of our two experimental studies with rhHNS in cynomolgus monkeys demonstrate important differences in the CNS distribution and exposure with IT dosing in comparison to IV dosing, as well as similarities in the systemic exposure of rhHNS dosed by the two methods. The pharmacokinetics and tissue penetration profiles of animals dosed with rhHNS was assessed by serum and CSF sampling at specified time points in Study A—phase 1 and Study B, by animal sacrifice and tissue harvest at specific time points in Study A—phase 2, and by positron emission tomography (PET) imaging using 124-iodinated rhHNS at specific time points in Study B.

IV dosing of rhHNS at 1.5 mg/kg in six animals in Study A and 1.0 mg in 4 animals in Study B did not result in appreciable penetration into the CSF or CNS tissues. In Study A, detection of rhHNS by ELISA showed mean CSF HNS levels were below LLOQ at the first time point and undetectable within 2 h. In Study B, IV dosing of iodinated rhHNS and detection by PET imaging showed an 8.9-fold lower C_max_ in the white matter compared to IT administration. In contrast, after IT administration at multiple doses (1.5, 4.5, and 14.5 mg in Study A; 3 mg in Study B), rapid tissue distribution and uptake of IT rhHNS throughout the CNS was observed. PET images in Study B showed strong signals of rhHNS presence in the cortex regions as early as 30 min post dose, although lower signal was observed in the deep, parenchymal regions of the brain including both white and grey matter areas. By 5 h post dose in Study B, clearance of rhHNS from the CSF ducts (septum pellucidum and third/fourth ventricles) and accumulation in the parenchyma and arachnoid was observed. 

As GAG build-up can also manifest, to a lesser extent, somatically in MPS IIIA, systemic exposure of rhHNS may be needed. Systemic exposure and distribution profiles of rhHNS were similar for IV and IT dosed rhHNS in Study A determined by ELISA and enzymatic assay, although peripheral organ exposures by IT administration were ~40–80% of the mean exposures following IV administration. In the kidneys, rhHNS levels were similar to those in the CNS tissues following IT administration, while levels in the liver were 10-fold higher than CNS tissues. In Study B, exposure in the heart and lungs was found to be low with IT rhHNS visualized by PET. 

PET imaging showed uptake and/or diffusion of IT rhHNS from CSF to blood to be rapid and appreciable in Study B at the (14.5 mg dose), as reflected in the high mean (SD) bioavailability estimate 47.6% (8.6%). The mean C_max_ (SD) of rhHNS in serum for this dose, 304 (87) ng/mL, was reached at 30.75 (22.16) h post dose, potentially providing therapeutic systemic exposure for reduction of GAG in urine, liver, and spleen. 

Recently, similar studies using intra-CSF injection or slow, continuous infusion in MPS IIIA dogs and in MPS IIIA mice were reported [26,27,28]. Beard et al. examined the MPS IIIA mouse brain tissue lesions and reported a superior outcome observed in slow enzyme-infused mice than in repeat bolus-treated animals [27]. On the other hand, King et al. reported significant reduction of primary substrate in brain tissues from dogs treated via spinal CSF bolus injection and also in animals treated with slow spinal CSF infusion [26,28]. The authors stated that the pump delivery of enzyme was less effective in reduction of secondary substrate in deeper regions of cerebral cortex. The authors further concluded that the continuous infusion of enzyme into MPS IIIA dog CSF did not reduce disease-based lesions as efficaciously as repeated cisternal or spinal CSF bolus treatment over the time-frame of these studies. The present study was conducted in wild-type cynomolgus monkeys, therefore, the comparison on effect in disease-derived brain lesions was not possible. However, our findings in this study provide quantitative evidence from the enzyme activity exposure levels in different brain regions. Our results showed that the intrathecally administered rhHNS was able to achieve and maintain above basal level (0.1 nmol/h/mg protein, unpublished data) up to 168 h post dose in cerebral cortex and up to 72 h post dose in white matter, brain structures, and nucleus. It supports the findings in King et al. of significantly lower substrate levels being achieved in dorsal cortex using cisternal bolus administration [26,28,29].

## 4. Materials and Methods

### 4.1. Study Designs

The pharmacokinetics profiles and biodistribution characteristics were assessed by two study approaches, Study A and Study B, which compared the systemic and CNS exposures and durations of rhHNS dosed IT and IV in cynomolgus monkeys. Dose response in serum and CSF and tissue penetration of rhHNS was determined in Study A using an enzyme linked immunosorbent assay (ELISA) and HNS activity assay. Study B assessed the dynamic uptake of rhHNS between the CNS and the systemic circulation using positron emission tomography (PET) imaging. The doses tested in each of these studies bracketed the range of anticipated potential clinical doses for human administration. 

Study A was a two-phase non-GLP study in 12 cynomolgus monkeys. Phase 1 consisted of 12 animals dosed at 1.5 mg/kg IV (*n* = 3) or 1.5 mg (*n* = 3), 4.5 mg (*n* = 3), and 13.5 mg (*n* = 3) IT administration of rhHNS. To determine the pharmacokinetics (PK) and biodistribution of rhHNS in the dosed animals, blood and CSF samples were collected at nine time points, from 5 min to 72 h post dose, for bioanalytical analysis. Phase 2 consisted of 12 animals dosed at 14.5 mg rhHNS IT. Blood and CSF samples were taken at six time points, from 1 h to 7 days post dose, immediately prior to animal sacrifice and tissue harvest.

Study B evaluated the PK properties and dynamic biodistribution of rhHNS in eight animals following 1.0 mg/kg IV or 3.0 mg IT administration using 124-iodinated (^124^I) rhHNS and positron emission tomography (PET) imaging. Blood samples for radioactivity and SEC HPLC measurement and images were taken every 30 min for 1 to 5 h, and at 24 and 48 h.

All animal studies were carried out in accordance with institutionally approved protocols at Association for Assessment and Accreditation of Laboratory Animal Care (AAALAC)–accredited facilities and were performed in accordance with the Guide for the Care and Use of Laboratory Animals (7th Edition, 1996, National Research Council, Washington, DC, USA). Study A was performed at Northern Biomedical Research, Inc. (NBR; Muskegon, MI, USA), an organization accredited by the Association for Assessment and Accreditation of Laboratory Animal Care. The study was approved by the Institutional Animal Care and Use Committee of NBR (cynomolgus monkey study protocol number 047-036 (12 January 2012). Study B was carried out at Shire, Lexington, MA, USA and approved by the Shire Nonclinical Development Institutional Animal Care and Use Committee under the Study ID HGT-1410-12-004 (3 June 2012).

In Study A, eight male and 10 female cynomolgus monkeys were surgically implanted with intrathecal lumbar catheters at the cisterna magna (CM) and lumbar spine. The CM catheter was used for CSF sample collection and the lumbar spine catheter was used for rhHNS dosing. Fourteen animals judged suitable for experimentation based on clinical sign, body weight, and catheter patency data were used for the study; six males and six females were dosed with rhHNS and two implanted dose-naïve male animals served as control animals. 

In Study A phase 1, six male and six female animals were randomly assigned by body weight into single dose treatment groups; 1.5 mg/kg IV, 1.5 mg IT, 4.5 mg IT, and 13.5 mg IT. The two highest dose treatment groups were animals from the two lower dose groups that underwent a washout period of 10–12 days before receiving a second dose of rhHNS at the higher doses. Blood and CSF samples were collected from the CM pre-dose and at specified intervals post dosing. After the last samples were collected, the animals were placed in phase 2 or dosed again in phase 1 after the washout. 

In Study A phase 2, six male and six female cynomolgus monkeys were assigned into 6 groups for scheduled tissue harvesting: 1 h, 4 h, 8 h; 1 day, 3 days, 7 days. Blood and CSF were collected from the CM prior to dosing and immediately prior to sacrifice. Post mortem, selected organs and tissues were harvested and saved for analysis.

In Study B, eight cynomolgus monkeys received radiolabelled ^124^I-rhHNS. Four animals received 1.0 mg/kg ^124^I-rhHNS administered IV through a temporary catheter installed in the great saphenous vein, and four animals received 3.0 mg of ^124^I-rhHNS administered IT through a pre-installed intrathecal drug delivery device (IDDD). Prior to ^124^I-rhHNS dosing IV or IT, animals were given subcutaneous non-radioactive iodine to suppress ^124^I uptake in the thyroid. PET images were acquired dynamically in the region of the injection site for 30 min post dose. Static images of the whole body, including the spine, were taken every 30 min out to 5 h post dose. Additionally, static images of the whole body were acquired at 24 and 48 h post dose.

### 4.2. Data Collection and Analysis

Pharmacokinetic analysis was performed at PKPD Bioscience Inc., using noncompartmental methods (WinNonlin™ Phoenix Version 6.1, Pharsight Corporation, Mountain View, CA, USA). Pharmacokinetic parameters in Study A were calculated from serum, CSF, and harvested tissues. Pharmacokinetic parameters in Study B were calculated from serum and PET images.

The PK analyses included test article concentrations for doses at specified time points, maximum observed concentration (C_max_), time of maximum observed concentration (T_max_), area under the concentration-time curve from time zero to the last sampling time at which concentrations were measurable (AUC_0-last_), terminal half-life (t1/2), and bioavailability (%F).

### 4.3. Study A Sample Collection and Handling

In Study A, approximately 1.25 mL of CSF was collected from the CM catheter of each animal for baseline total cell count and chemistry analysis 5 to 7 days post catheter implantation surgery. In phase 1, approximately 0.1 mL CSF samples were collected from the CM catheter immediately predose and at 5 min, 15 min, 30 min, 1, 2, 4, 8, 24, 48, and 72 h post dose. In phase 2, approximately 0.25 mL CSF samples were collected from the CM catheter predose and immediately prior to sacrifice. Additionally, CSF was collected from two implanted, dose-naïve male animals by CM tap for control sample analysis. A CM spinal tap was performed if the CM catheter was found to be blocked. CSF samples for chemistry and PK analysis were stored at −60 °C or colder until they were analyzed. The chemistry analysis included albumin, calcium, chloride, glucose, inorganic phosphate, potassium, sodium, and total protein determinations. 

In Study A, blood samples (~1 mL) were collected from a peripheral vein for serum test article analysis. For phase 1, samples were taken predose and 5 min, 15 min, 30 min, 1, 2, 4, 8, 24, 48, and 72 h post dose. In phase 2, samples were taken predose and immediately prior to sacrifice and tissue harvesting. For control samples, blood samples (6 mL) were collected from two implanted, dose-naïve male animals prior to sacrifice and tissue harvesting. Once collected, the blood sample tubes were centrifuged; the serum was transferred to labelled vials, and stored at −60 °C or below.

Post mortem methods used in phase 2 of Study A were carried out in the following manner; prior to tissue harvesting, animals were sedated with 8 mg/kg of ketamine HCl dosed intramuscularly, maintained on an isoflurane/oxygen mixture, and provided with an IV bolus of heparin sodium, 200 IU/kg. The animals were then perfused via the left cardiac ventricle with 0.001% sodium nitrite in saline. One male and one female animal were sacrificed at each time point: 1 h, 4 h, 8 h, 1 day, 3 days, and 7 days for tissue harvesting. Specifically, the following tissues were used for analysis: Cerebral Cortex (superficial, temporal, and frontal cortex), CNS Nucleus (caudate nucleus, hypothalamus, and thalamus), CNS Structures (cerebellum, corpus callosum, and hippocampus), White Matter (deep and superficial), Spinal Cord tissues, Liver (both lobes), and kidney (both lobes). White matter tissues were sampled from both “superficial” and “deep” regions. “Superficial” samples were taken from the top 3 mm or less of brain tissue, and “deep” samples were taken from a depth of 3 mm or greater.

In Study A, bioanalytical sample analysis was conducted by WIL Research (Skokie, IL, USA) using validated ELISA and HNS enzymatic activity assay for measurement of serum and CSF HNS concentrations and HNS activity in tissue homogenates, respectively. The standard sandwich ELISA was used to measure rhHNS in serum and CSF. Briefly, the rhHNS in sample was captured by an anti-rhHNS monoclonal antibody (Ab) immobilized on a 96 well plate and detected by a biotin-anti-rhHNS polyclonal Ab. Following incubation with streptavidin-HRP then TMB, the reaction was stopped by sulfuric acid. The rhHNS concentrations in test samples were calculated based on an rhHNS calibration curve in the same assay. The paired Abs for HNS ELISA were generated against rhHNS, but might cross-react with endogenous HNS in cynomolgus (cyno) monkey serum and CSF. The HNS results from 8/10 lots of individual cyno monkey serum samples and 10/10 lots of individual cyno monkey CSF samples were BLQ during assay validation. The HNS activity in tissue homogenate was measured by a two-step activity using HNS substrate 4-methylumbelliferyl-α-d-*N*-sulphoglucosaminide, Na, and secondary enzyme α-glucosidase [30]. The final product 4-methylumbelliferone (4-MU) concentrations were calculated based on the 4-MU calibration curve in the same assay. The HNS activity (nmol/h/mL) was normalized to the total protein concentration of tissue homogenate and reported as nmol/h/mg protein. The activity assay detects HNS activity from both human HNS and endogenous monkey HNS. The cross reactivity to endogenous cyno monkey HNS do not impact PK modeling, since the baseline samples prior dosing or from control untreated cyno monkey were collected and evaluated by both HNS ELISA and activity assays.

In Study A, immunohistochemical analysis was used to evaluate cellular biodistribution of intrathecal injected rhHNS protein using a mouse monoclonal anti-human rhHNS (2C7) antibody. Briefly, tissue slides were deparaffinized and retrieved in citrate buffer, then incubated with the mouse anti-rhHNS primary antibody. Following incubation, a secondary anti-mouse biotinylated IgG was applied. After additional incubation, avidin and biotinylated horseradish peroxidase macromolecular complex was added for another incubation. Finally, the slides were incubated in peroxidase substrate H_2_O_2_ and chromogen DAB (diaminobenzidine) solution, and nuclei were counterstained with hematoxylin. rhHNS positive cells were identified by brown staining; nuclei were stained blue.

### 4.4. Study B Sample Collection and Imaging

In Study B, the rhHNS test article analysis in serum was carried out by size exclusion High Performance Liquid Chromatography (HPLC) that determines the disassociation of ^124^I radiolabel used to construct individual blood concentration-time profiles of rhHNS based on sample radioactivity and specific activity of ^124^I-rhHNS. Blood samples were taken at the start of each whole-body imaging study following IV and IT administration.

The whole-body PET images were composed of acquired section images with a 12 mm overlap between sections. Reconstructed images were analyzed to obtain numerical data from manually segmented three-dimensional regions of interest (typically 0.2 to 5 mL in volume) containing organs and tissues of interest. Extracted radioactivity data (expressed in nCi per mL) were converted into protein concentration and percentage of injected dose per mL (%ID/mL). Corrections typical for PET data evaluation, such as transmission correction, radionuclide decay, and scatter, were applied as required. 

## 5. Conclusions

When rhHNS was administered intravenously, rhHNS levels were low to non-detectable in CSF and CNS tissues. When intrathecally dosed, rhHNS showed rapid uptake from the spinal cord CSF into CNS tissues with peak concentrations occurring within the first 24 h of administration. At 7 days, rhHNS was still detectable in the CNS and spinal cord. Penetration into grey matter and cortex was 3–4 times greater than concentrations in white matter and deeper parenchymal regions, suggesting some limitations of this ERT strategy. PET imaging confirmed the distribution of IT-dosed rhHNS from CSF to blood can be rapid and appreciable, and the systemic exposure and distribution profiles of rhHNS was similar for IV and IT dosed rhHNS. These findings provide evidence that intrathecal dosing of rhHNS delivers the replacement enzyme to therapeutically relevant tissues. 

## Figures and Tables

**Table 1:** Study Design for Study A-1, A-2, and Study B.

Study (-Phase)	*n* Animals (M/F)	Dose(s) (mg/kg IV, mg IT)	Samples/Data Collected	Time Points	Analysis Methods
A-1	6 animals (3M/4F)	1.5 mg/kg IV	Blood, CSF	5 min, 15 min, 30 min, 1 h, 2 h, 4 h, 8 h, 1 day, 2 days, 3 days post dose	ELISA
A-1	6 animals per dose (3M/4F)	1.5 mg, 4.5 mg, 13.5 mg IT	Blood, CSF	5 min, 15 min, 30 min, 1 h, 2 h, 4 h, 8 h, 1 day, 2 days, 3 days post dose	ELISA
A-2	2 animals per time point (1M/1F)	14.5 mg IT	Blood, CSF, Tissues	predose and 1 h, 4 h, 8 h, 1 day, 3 days, 7 days post dose	ELISA, enzyme activity
B	4 animals (2M/2F)	1 mg/kg IV	Blood, whole body PET	*t* = 0 and every 30 min 1–5 h, then at 1 day and 2 days	Radioactivity HPLC, PET
B	4 animals (2M/2F)	3 mg IT	Blood, whole body PET	*t* = 0 and every 30 min 1–5 h, then at 1 day and 2 days	Radioactivity HPLC, PET

Intravenous (IV); intrathecal (IT); cerebral spinal fluid (CSF); enzyme linked immunosorbent assay (ELISA); high performance liquid chromatography (HPLC), positron emission tomography (PET).

**Table 2:** Selected mean pharmacokinetics (PK) parameters from Tissue Analysis of rhHNS dosed IT at 14.5 mg.

Tissue	T_1/2_ (h)	T_max_ (h)	C_max_ (nmol/h/mg * Protein)	AUC_0-last_ (nmol/h/mg * Protein)
Spinal cord	24.1	19.5	104	6570
Cerebral Cortex	32.7	24	5.83	309
CNS Nucleus	58.2	2.2	0.635	14.0
CNS Structures	35.4	12.5	2.56	81.2
White Matter	35.2	6.8	1.36	33.6
Liver	29.5	8	9.20	441
Kidney	65.8	4	2.10	134

* C_max_ and AUC_0-last_ values from tissue homogenates from 4 animals are expressed as HNS activity with the units nmol/h/mg. Cerebral Cortex (superficial, temporal, and frontal cortex); CNS Nucleus (caudate nucleus, hypothalamus, and thalamus); CNS structures (cerebellum, corpus callosum, and hippocampus); White Matter (deep and superficial). Note: Table rows are color coded to match the line graph colors in Figure 3 and Figure 4 for ease of reference.

## Supplementary Materials

Supplementary materials can be found at www.mdpi.com/1422-0067/18/12/2594/s1.

## Abbreviations

ERT	Enzyme replacement therapy
rhHNS	Recombinant human heparan *N*-sulfatase
HNS	Heparan *N*-sulfatase
MPS IIIA	Mucopolysaccharidosis III type A
IT	Intrathecal administration
IV	Intravenous administration
ELISA	Enzyme linked immunosorbent assay
PET	Positron emission tomography
PK	Pharmacokinetics
CSF	Cerebral spinal fluid
CNS	Central nervous system
GAG	Glycoaminoglycan

## References

[1] Froissart R., Maire I. Mucopolysaccharidosis Type 3. http://www.orpha.net/consor/cgi-bin/OC_Exp.php?lng=EN&Expert=581.

[2] Valstar M.J., Marchal J.P., Grootenhuis M., Colland V., Wijburg F.A. (2011). Cognitive development in patients with mucopolysaccharidosis type iii (sanfilippo syndrome). Orphanet J. Rare Dis..

[3] Shapiro E.G., Nestrasil I., Delaney K.A., Rudser K., Kovac V., Nair N., Richard C.W., Haslett P., Whitley C.B. (2016). A prospective natural history study of mucopolysaccharidosis type iiia. J. Pediatr..

[4] Pfeifer R.W., Felice B.R., Boyd R.B., Butt M.T., Ruiz J.A., Heartlein M.W., Calias P. (2012). Safety evaluation of chronic intrathecal administration of heparan n-sulfatase in juvenile cynomolgus monkeys. Drug Deliv. Transl. Res..

[5] Jones S.A., Breen C., Heap F., Rust S., de Ruijter J., Tump E., Marchal J.P., Pan L., Qiu Y., Chung J.K. (2016). A phase 1/2 study of intrathecal heparan-n-sulfatase in patients with mucopolysaccharidosis iiia. Mol. Genet. Metab..

[6] Muenzer J., Hendriksz C.J., Fan Z., Vijayaraghavan S., Perry V., Santra S., Solanki G.A., Mascelli M.A., Pan L., Wang N. (2016). A phase i/ii study of intrathecal idursulfase-it in children with severe mucopolysaccharidosis ii. Genet. Med..

[7] Burton B. Orphanet: Mucopolysaccharidosis Type 2, Severe Form. http://www.orpha.net/consor/cgi-bin/OC_Exp.php?lng=EN&Expert=217085.

[8] Felice B.R., Wright T.L., Boyd R.B., Butt M.T., Pfeifer R.W., Pan J., Ruiz J.A., Heartlein M.W., Calias P. (2011). Safety evaluation of chronic intrathecal administration of idursulfase-it in cynomolgus monkeys. Toxicol. Pathol..

[9] Calias P., Papisov M., Pan J., Savioli N., Belov V., Huang Y., Lotterhand J., Alessandrini M., Liu N., Fischman A.J. (2012). CNS penetration of intrathecal-lumbar idursulfase in the monkey, dog and mouse: Implications for neurological outcomes of lysosomal storage disorder. PLoS ONE.

[10] Xie H., Chung J.K., Mascelli M.A., McCauley T.G. (2015). Pharmacokinetics and bioavailability of a therapeutic enzyme (idursulfase) in cynomolgus monkeys after intrathecal and intravenous administration. PLoS ONE.

[11] Gliddon B.L., Hopwood J.J. (2004). Enzyme-replacement therapy from birth delays the development of behavior and learning problems in mucopolysaccharidosis type iiia mice. Pediatr. Res..

[12] de Ruijter J., Valstar M.J., Wijburg F.A. (2011). Mucopolysaccharidosis type iii (sanfilippo syndrome): Emerging treatment strategies. Curr. Pharm. Biotechnol..

[13] Fu H., Dirosario J., Killedar S., Zaraspe K., McCarty D.M. (2011). Correction of neurological disease of mucopolysaccharidosis iiib in adult mice by raav9 trans-blood-brain barrier gene delivery. Mol. Ther. J. Am. Soc. Gene Ther..

[14] Murrey D.A., Naughton B.J., Duncan F.J., Meadows A.S., Ware T.A., Campbell K., Bremer W.G., Walker C., Goodchild L., Bolon B. (2014). Feasibility and safety of systemic raav9-hnaglu delivery for treating mps iiib: Toxicology, bio-distribution and immunological assessments in primates. Hum. Gene Ther. Clin. Dev..

[15] Truxal A.E., Slack C.C., Gomes M.D., Vassiliou C.C., Wemmer D.E., Pines A. (2016). Nondisruptive dissolution of hyperpolarized (129)xe into viscous aqueous and organic liquid crystalline environments. Angew. Chem..

[16] Truxal K.V., Fu H., McCarty D.M., McNally K.A., Kunkler K.L., Zumberge N.A., Martin L., Aylward S.C., Alfano L.N., Berry K.M. (2016). A prospective one-year natural history study of mucopolysaccharidosis types iiia and iiib: Implications for clinical trial design. Mol. Genet. Metab..

[17] Sorrentino N.C., Fraldi A. (2016). Brain targeting in mps-iiia. Pediatr. Endocrinol. Rev. PER.

[18] Cox T., Lachmann R., Hollak C., Aerts J., van Weely S., Hrebicek M., Platt F., Butters T., Dwek R., Moyses C. (2000). Novel oral treatment of gaucher’s disease with n-butyldeoxynojirimycin (ogt 918) to decrease substrate biosynthesis. Lancet.

[19] Elstein D., Hollak C., Aerts J.M., van Weely S., Maas M., Cox T.M., Lachmann R.H., Hrebicek M., Platt F.M., Butters T.D. (2004). Sustained therapeutic effects of oral miglustat (zavesca, n-butyldeoxynojirimycin, ogt 918) in type i gaucher disease. J. Inherit. Metab. Dis..

[20] Schiffmann R., Fitzgibbon E.J., Harris C., DeVile C., Davies E.H., Abel L., van Schaik I.N., Benko W., Timmons M., Ries M. (2008). Randomized, controlled trial of miglustat in gaucher’s disease type 3. Ann. Neurol..

[21] Bennett L.L., Mohan D. (2013). Gaucher disease and its treatment options. Ann. Pharmacother..

[22] Parini R., Rigoldi M., Tedesco L., Boffi L., Brambilla A., Bertoletti S., Boncimino A., Del Longo A., De Lorenzo P., Gaini R. (2015). Enzymatic replacement therapy for hunter disease: Up to 9 years experience with 17 patients. Mol. Genet. Metab. Rep..

[23] Garbuzova-Davis S., Mirtyl S., Sallot S.A., Hernandez-Ontiveros D.G., Haller E., Sanberg P.R. (2013). Blood-brain barrier impairment in mps iii patients. BMC Neurol..

[24] Calias P., Banks W.A., Begley D., Scarpa M., Dickson P. (2014). Intrathecal delivery of protein therapeutics to the brain: A critical reassessment. Pharmacol. Ther..

[25] Voznyi Y.V., Keulemans J.L., van Diggelen O.P. (2001). A fluorimetric enzyme assay for the diagnosis of mps ii (hunter disease). J. Inherit. Metab. Dis..

[26] King B., Hassiotis S., Rozaklis T., Beard H., Trim P.J., Snel M.F., Hopwood J.J., Hemsley K.M. (2016). Low-dose, continuous enzyme replacement therapy ameliorates brain pathology in the neurodegenerative lysosomal disorder mucopolysaccharidosis type iiia. J. Oneurochem..

[27] Beard H., Hassiotis S., Luck A.J., Rozaklis T., Hopwood J.J., Hemsley K.M. (2016). Continual low-dose infusion of sulfamidase is superior to intermittent high-dose delivery in ameliorating neuropathology in the MPS IIIA mouse brain. JIMD Rep..

[28] King B., Marshall N., Beard H., Hassiotis S., Trim P.J., Snel M.F., Rozaklis T., Jolly R.D., Hopwood J.J., Hemsley K.M. (2015). Evaluation of enzyme dose and dose-frequency in ameliorating substrate accumulation in MPS IIIA huntaway dog brain. J. Inherit. Metab. Dis..

[29] King B., Setford M.L., Hassiotis S., Trim P.J., Duplock S., Tucker J.N., Hattersley K., Snel M.F., Hopwood J.J., Hemsley K.M. (2016). Low-dose, continual enzyme delivery ameliorates some aspects of established brain disease in a mouse model of a childhood-onset neurodegenerative disorder. Exp. Neurol..

[30] Karpova E.A., Voznyi Ya V., Keulemans J.L., Hoogeveen A.T., Winchester B., Tsvetkova I.V., van Diggelen O.P. (1996). A fluorimetric enzyme assay for the diagnosis of sanfilippo disease type a (MPS IIIA). J. Inherit. Metab. Dis..

