# Supplementary figures and images for: Whole Body and CNS Biodistribution of rhHNS in Cynomolgus Monkeys after Intrathecal Lumbar Administration: Treatment Implications for Patients with MPS IIIA
Source: Int J Mol Sci. 2017 Dec 1;18(12):2594. doi: 10.3390/ijms18122594 (PMC5751197; doi:10.3390/ijms18122594)

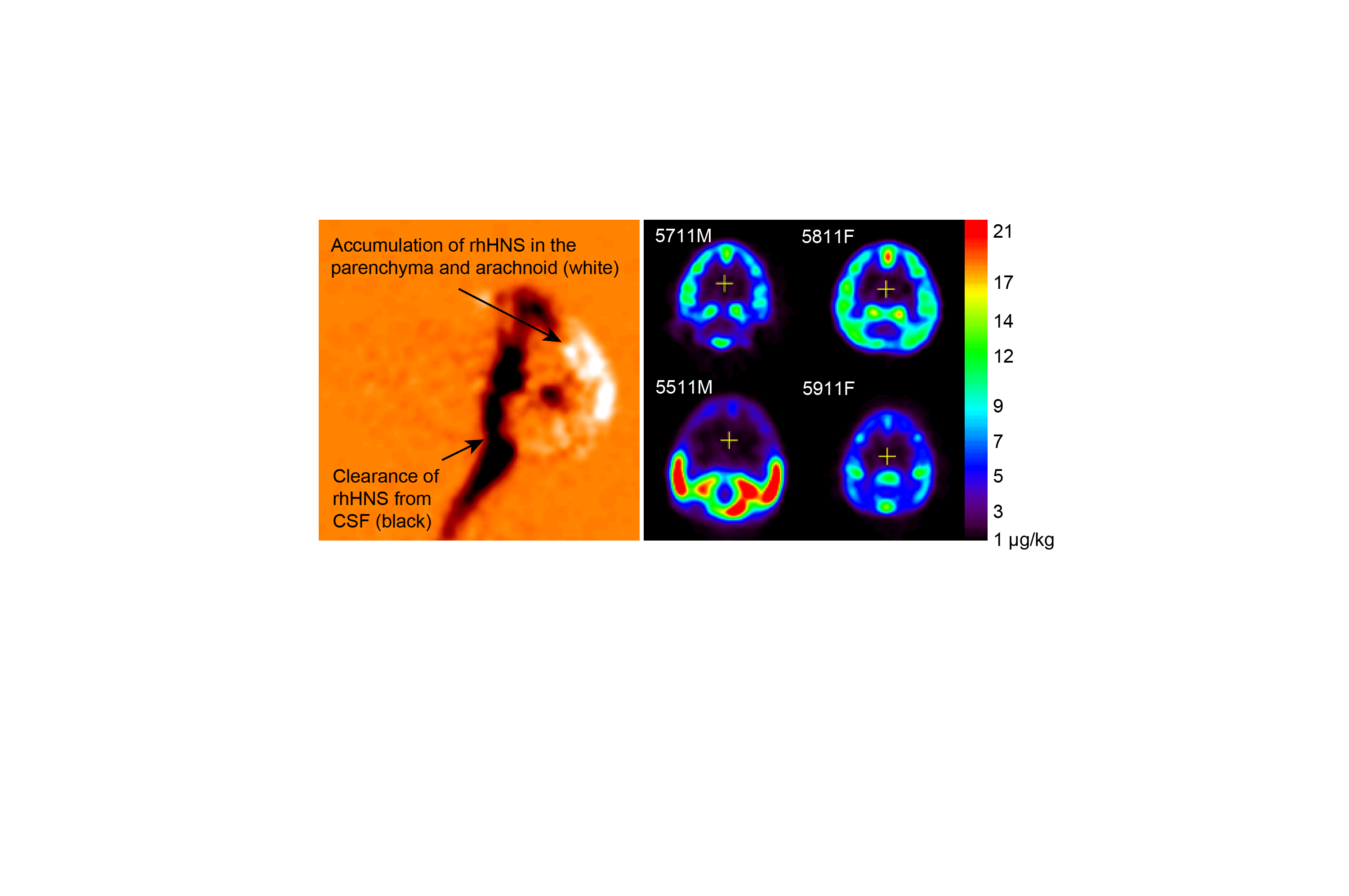

Supplement: Supplementary file 1 [file ijms-18-02594-s001.zip › ijms-235551-Supplementary Figure S4 Cerebral Uptake.png]

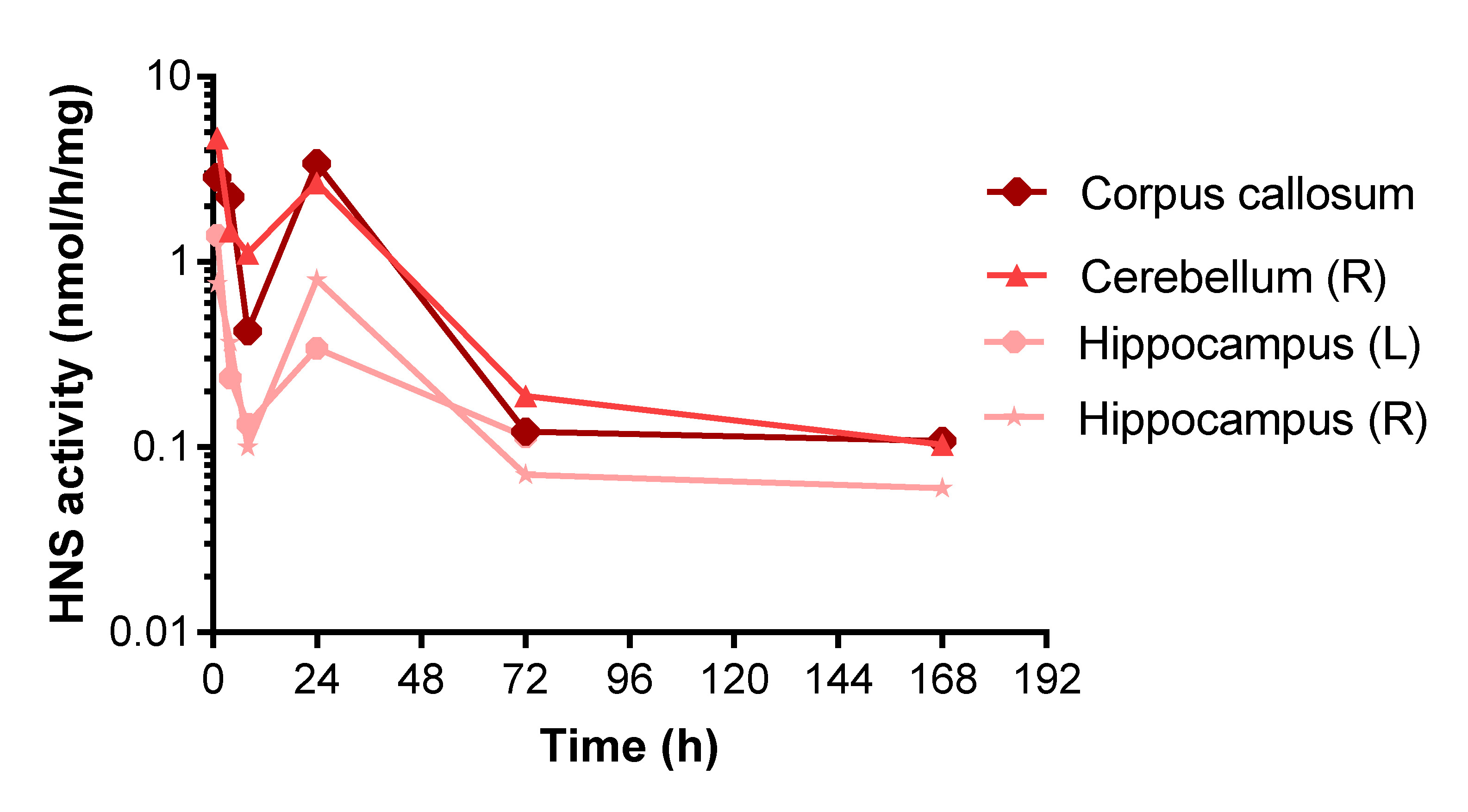

Supplement: Supplementary file 1 [file ijms-18-02594-s001.zip › ijms-235551-Supplementary Figure S3 Activity of HNS Cerebellum.jpg]

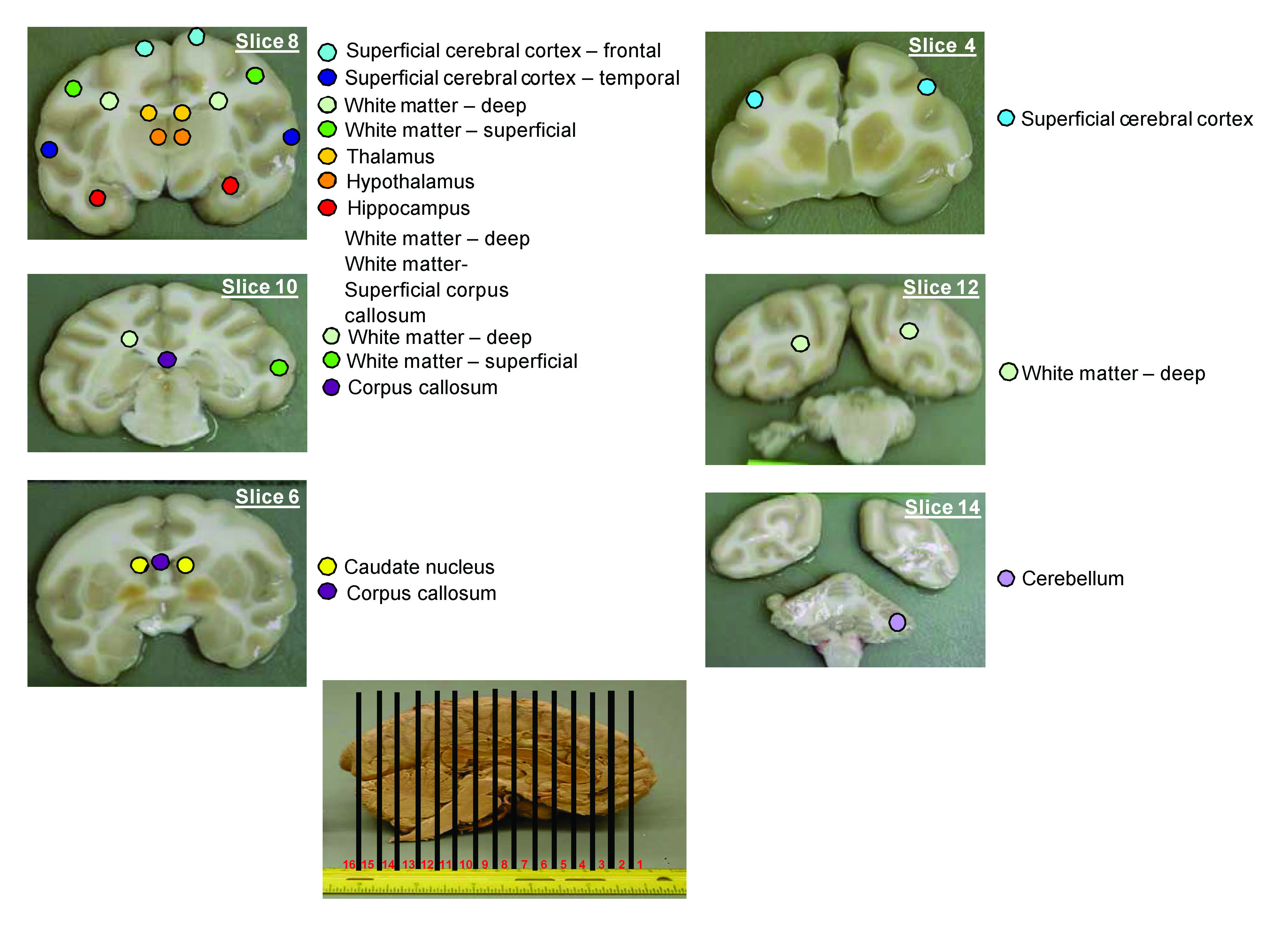

Supplement: Supplementary file 1 [file ijms-18-02594-s001.zip › ijms-235551-Supplementary Figure S2 Brain Tissue Map.jpg]

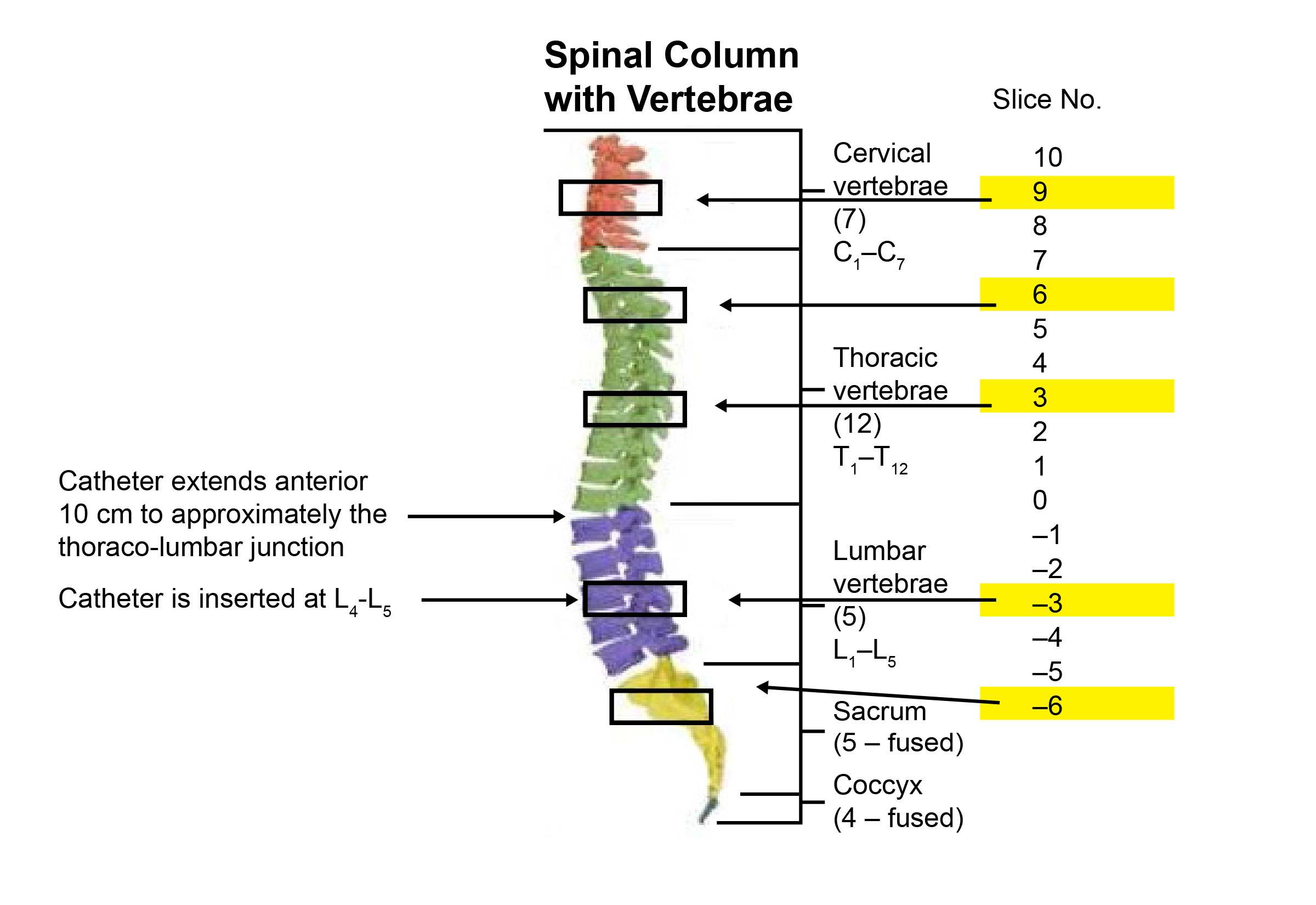

Supplement: Supplementary file 1 [file ijms-18-02594-s001.zip › ijms-235551-Supplementary Figure S1 Spinal Cord Sections.jpg]
